# Will Specialists Become Extinct?

**Published:** 1893-07-22

**Authors:** 


### The Hospital

An Institutional Journal of

Science, Medicine, Nursing, and Philanthropy.

*For Hospitals, Asylums, Infirmaries, Dispensaries, Medical Practitioners, Students, Nurses, and the Charitable Public.*

Vol. XIV.—No. 356.] July 22, 1893.

### Will Specialists Become Extinct?

In the "Essays" just published of the late Sir Morell Mackenzie, it is contended that there must be an "inevitable disappearance of the general physician in the struggle for existence." Concerning this the *Spectator* remarks, "If this is to happen, we can only say it is much to be regretted. However, nine-tenths of mankind will continue to be treated by the general practitioner, of whom, we take it, the general physician is only a fine variety." The question of the future of specialism is manifestly of importance to the laity, as it certainly is of very commanding interest to the medical profession.

The general physician, that is the consultant of historical centuries, thought Sir Morell Mackenzie, must, in the struggle for existence, inevitably disappear. In our humble judgment the exact opposite is what will happen. It is the specialist as we know him, who is bound by the laws of evolution and the imperatives of supply and demand, to disappear, and to vanish as if he had never been. What is the medical specialist of to-day? In order to understand him well, let us compare him with the clerical specialist, or with the specialist of law. The medical specialist is a specialist pure and simple. He fixes himself upon one small part of the human organism, and it is his pride to deal exclusively with that, and nothing else. Is he a specialist for the eye; and does the eye demand iron, general tonics, food and fresh air? He does not prescribe the iron, the food, and the fresh air; he sends his patient to the general physician. Is he a dentist? Does the state of his patient's teeth demand alteratives and the improvement of the digestion? He does not know how to prescribe an alterative; and for the digestion he has nothing but contempt. It is his pride to be not a surgeon but a specialist; not a physician, but a specialist.

Now turn to the clerical specialist, to the commentator, the philologist, the historical critic, the collater of manuscripts, the philosophical expositor: to a Lightfoot, a Driver, an Ewald, a Fairbairn, a Robertson Smith. Every one of these is a specialist, but all of them without exception are working clergymen, vicars, bishops, or University professors, and every one of them finds infinitely more satisfaction in the general advancement of true religion than in the undue prominence of his own particular specialty. There is true comprehensiveness of mind here. The clerical specialist who is also a bishop is a bishop rather than a specialist. He has found in himself some special faculty of mind, or he has found in his circumstances some special opportunity, and of these he has taken advantage in order to increase the general sum of human knowledge or the general power of mankind to cope with its infinitely varied difficulties. But he has never said, I am a philologist rather than a clergyman, an expositor rather than a bishop. He has always made it manifest that he is a general clergyman, with a profound special capacity and knowledge, and that both his general and his special knowledge are equally at the service of man.

But, in truth, the history of special hospitals shows us exactly what will happen to specialist practitioners. The day of the special hospital is over. No understanding person nowadays thinks of founding a special hospital, and for this reason, that every rational specialty is pursued in the special departments of the general hospitals. The specialist will inevitably follow in the train of his special hospital. What, indeed, do we see already? Do we not see that the very specialist himself, when he has the opportunity, repudiates his special hospital in order to become attached to the special department of a general hospital? What will be the next step? He will gradually become ashamed of the narrow and limited appellation of aurist, or oculist, or dentist, and will aspire to the far more dignified and honoured title of physician or surgeon.

The particular agency of development which will squeeze out the "pure" specialist, as he calls himself —"the half-cultured proprietor of one idea, or the conjuror of one trick," as some might prefer to call him—will be the highly educated general practitioner. The practitioner of the present day aspires to be a philosophic thinker, a master of the whole of his business. Why, he argues, should I not know all about the ear, as well as all about the tongue? Why should I not master the knowledge of the structure and diseases of the eye as well as that of the structure and diseases of the stomach or the liver, the kidneys or the spleen? As a matter of fact, the more competent of general practitioners are already familiar with the normal eye and ear, and with the commoner abnormalities and their management. Moreover, every medical school which claims to give a complete medical education, and every student who has the honourable ambition to be a "full, ready, and complete" practitioner, in Bacon's phraseology, enters for separate courses on the eye, ear, and other specialties as a part of his ordinary training. The day, indeed, is close at hand when every practitioner will be compelled to master, at least the elements of every specialty, whether he likes it or not, before he will be allowed to pass any of the qualifying examinations for a diploma in medicine or surgery.

If the writer of the *Spectator's* article were asked for the reason why he deplored the possible extinction of the general physician, the answer, we imagine, would be by no means long delayed. He would say that the specialist, being by the nature of the case essentially a man of one idea, and that of the most limited range, must inevitably lower the standard of culture and capacity throughout the whole medical profession. Let us recur for a moment to our clerical comparison. What would be thought, for example, of a Hebrew moralist who should concentrate the whole of his instructions upon a single commandment of the Decalogue, instead of illustrating and enforcing the equally important obligations of the whole ten? Or, what value should we set upon a Christian divine who should insist upon making a specialty of the first precept of the Sermon on the Mount, and should send troubled souls to a neighbouring clergyman if they happened to desire instruction in any other portion of the New Testament?

The cases of the doctor and the clergyman, it seems to us, are strictly parallel. Specialism, which consists in special devotion to a limited area of work for the purposes of special elucidation is one thing, and is not only honourable, but absolutely essential to progress. But specialism which consists in the claim to do only one thing in practice, and that a small thing, is quite another matter, and is a hobby which, when ridden too much, carries its riders dangerously near the borderland of pettifogging and pedantry. Besides, is it not a proposition which cannot be questioned that the man who is a specialist and not a generalist as well cannot really be a competent specialist? Why is it that specialists and special hospitals have lost so much ground in the general esteem even within the brief space of the last five years? It is simply because their specialism has consisted in special claims, and not in specially successful results of treatment. It is a fact well known to every doctor that when the competent general physician has confessed his inability to do anything more for his patient there is distressingly little hope in the specialist.

There are in medicine two legitimate specialties, and these two are the "operating" specialty and the "prescribing" specialty. One medical student is of an objective and mechanical turn of mind. He, when opportunity serves, will become a surgeon; and if his education be complete, he will cover the whole ground of operative surgery. Another will be of an abstract, contemplative, and sympathetic turn of mind; and he will be a general physician. Those who have special opportunities or special aptitudes will still devote special labour to special organs, diseases, and methods of treatment. But they will claim for themselves the honourable titles and work of the physician and the surgeon, and not the poor prominence of the specialist. This, it seems to us, is the true line of medical development, and all young practitioners and medical students will do well to lay their account with it.

